# A risk prediction model for overall and Grade C anastomotic leakage after rectal cancer surgery

**DOI:** 10.3389/fonc.2026.1741353

**Published:** 2026-04-10

**Authors:** Wenpeng Wang, Shan Gao, Wenhui Zhao, Liancheng Li, Duo Yun, Jiefu Wang

**Affiliations:** 1 Department of Colorectal Oncology, Tianjin Medical University Cancer Institute and Hospital, National Clinical Research Center for Cancer, Tianjin’s Clinical Research Center for Cancer, Tianjin Key Laboratory of Digestive Cancer, Tianjin, China; 2 Department of Immunology, Tianjin Medical University Cancer Institute and Hospital, National Clinical Research Center for Cancer, Key Laboratory of Cancer Prevention and Therapy, Tianjin, Tianjin’s Clinical Research Center for Cancer, Tianjin, China; 3 North Campus Acute Care Ward (Geriatric Critical Care Unit), First Teaching Hospital of Tianjin University of Traditional Chinese Medicine, National Clinical Research Center for Chinese Medicine Acupuncture and Moxibustion, Tianjin University of Traditional Chinese Medicine, Tianjin, China; 4 Cancer Center, Beijing Friendship Hospital, Capital Medical University, Beijing, China

**Keywords:** anastomotic leak, grade C leakage, prediction model, rectal neoplasms, risk factors

## Abstract

**Objective:**

This study aimed to identify predictors of anastomotic leakage (AL), including Grade C AL, after rectal cancer surgery and to establish a risk prediction model for clinical risk stratification.

**Methods:**

A retrospective study was conducted on rectal cancer patients who underwent anterior resection (AR) at Tianjin Medical University Cancer Institute and Hospital between November 2020 and November 2024. Clinicopathological variables were analyzed, and multivariable logistic regression was applied to construct predictive models for overall and Grade C anastomotic leakage.

**Results:**

A total of 901 rectal cancer patients were included, with an AL incidence of 8.9% (80/901) and Grade C AL occurring in 4.7% (42/901). Multivariable analysis identified postoperative numerical rating scale (NRS) pain score (OR = 9.556; 95% CI, 6.014-15.184; p < 0.001), neoadjuvant chemoradiotherapy (NACRT) (OR = 3.070; 95% CI, 1.525-6.182; p = 0.002), intersphincteric resection (ISR) (OR = 4.928; 95% CI, 1.340-18.126; p = 0.016), intestinal obstruction (OR = 2.926; 95% CI, 1.105-7.748; p = 0.031), tumor size (OR = 2.238; 95% CI, 1.239-4.042; p = 0.008), operative time (OR = 2.416; 95% CI, 1.092-5.349; p = 0.030), diverting stoma (OR = 0.124; 95% CI, 0.031-0.491; p = 0.003), and gender (female vs. male) (OR = 0.410; 95% CI, 0.220-0.765; p = 0.005) as independent predictors of overall AL. For Grade C AL, NRS pain score (OR = 6.563; 95% CI, 2.565-16.791; p < 0.001) and NACRT (OR = 7.534; 95% CI, 2.012-28.216; p = 0.003) were significant predictors. The nomogram demonstrated strong discrimination, with C-statistics of 0.872 for overall AL and 0.817 for Grade C AL. NRS pain score achieved the highest individual predictive performance (AUC = 0.812 for overall AL; 0.759 for Grade C AL). Combined models integrating NRS with other variables further improved accuracy (AUC = 0.856 for overall AL; 0.817 for Grade C AL). Calibration curves showed excellent agreement between predicted and observed outcomes.

**Conclusion:**

We developed a risk prediction model for AL after rectal cancer surgery using preoperative, intraoperative, and early postoperative variables. The NRS pain score was the strongest predictor, and any unexplained rise in pain should raise suspicion of impending AL. This model offers a practical tool for early postoperative risk stratification and enhanced monitoring in high-risk patients.

## Introduction

Anastomotic leakage (AL) remains one of the most serious complications after rectal cancer surgery, with Grade C AL representing the most severe form and being associated with high rates of sepsis, reoperation, and mortality, thereby exerting a substantial impact on patient outcomes and healthcare resources (1–3). Despite advancements in surgical techniques, such as total mesorectal excision (TME) and minimally invasive approaches including robotic-assisted surgery, the incidence of AL remains at 3–21%, with Grade C cases constituting a significant proportion of severe events (4–8).

In recent years, numerous studies have explored potential risk factors for AL following rectal cancer surgery. These studies have investigated a range of factors, including patient-related characteristics, tumor features, and surgical techniques. However, the results have been inconsistent, possibly due to heterogeneous study populations, differences in surgical approaches, and evolving treatment protocols. Several key studies have highlighted significant risk factors for AL, including advanced age, male gender, tumor location, and the use of neoadjuvant chemoradiotherapy (NACRT). Nevertheless, the role of NACRT in AL development remains controversial, with conflicting evidence regarding its impact (9–11). In addition, postoperative factors, such as the numerical rating scale (NRS) for pain, have recently been proposed as potential early indicators of AL, though their predictive value requires further investigation (12).

Therefore, this retrospective single-center study aimed to identify independent predictors of AL, with a particular focus on Grade C AL, after rectal cancer surgery. To achieve a comprehensive risk assessment that reflects the entire perioperative process, we analyzed a spectrum of variables collected at different time points. These included preoperative baseline clinical characteristics, intraoperative surgical details, and early postoperative variable such as the NRS for pain. Specifically, to evaluate the potential of pain as an early bedside indicator, we utilized the NRS score recorded on the day prior to the confirmed diagnosis of AL. By integrating predictors from these distinct phases—spanning from before surgery to the early postoperative period—we sought to develop a risk prediction model that captures both baseline susceptibility and early warning signals. This approach aims to support dynamic risk stratification and optimize perioperative management strategies, moving beyond static preoperative predictions to incorporate the evolving clinical reality of the patient.

## Patients and methods

### Study design and ethical approval

This retrospective cohort study was conducted at Tianjin Medical University Cancer Institute and Hospital between November 2020 and November 2024. The study protocol adhered to the ethical principles of the Declaration of Helsinki and was approved by the Institutional Review Board of Tianjin Medical University Cancer Institute and Hospital (No. bc20253331). Given the retrospective design, the requirement for written informed consent was waived.

### Patient selection criteria

Patients with rectal cancer who underwent anterior resection (AR), either via laparoscopic or open approach, were screened. Inclusion criteria were: (1) histologically confirmed rectal adenocarcinoma within 15 cm of the anal verge (AV); (2) standardized TME performed with curative intent; and (3) availability of complete clinicopathological data. Exclusion criteria were: (1) multivisceral resection; (2) modified resection for locally advanced or unresectable disease; (3) abdominoperineal resection (APR) or Hartmann procedure; and (4) synchronous colorectal malignancies.

### Data collection

Demographic, clinical, surgical, and pathological data were retrospectively retrieved from the institutional electronic medical record system. The study assessed the association of these variables with AL. Demographic variables included sex, age, and body mass index (BMI). Clinical variables encompassed tumor distance from the AV, preoperative albumin level, presence of intestinal obstruction, NACRT, and American Society of Anesthesiologists (ASA) classification. Surgical-pathological variables comprised operative time, intraoperative blood loss, surgical approach, anastomotic technique, diverting stoma creation, intersphincteric resection (ISR), tumor size, pathological TNM stage, tumor deposits, and postoperative NRS pain score.

Postoperative pain was evaluated using the NRS, an 11-point scale ranging from 0 (“no pain”) to 10 (“worst pain imaginable”) (13). Pain scores were recorded daily for all patients throughout their postoperative hospital stay. For patients who developed AL, the NRS score recorded on the day prior to the confirmed diagnosis was used for analysis, as it was considered to have greater predictive value. For patients without AL, the highest NRS score recorded during hospitalization was used as a comparator to represent the peak pain experience in uncomplicated patients.

Total neoadjuvant therapy (TNT) was selectively administered to patients with locally advanced rectal cancer presenting with specific high-risk features on pretreatment MRI, as determined by multidisciplinary team (MDT) discussion. These features included: clinical T4 stage, extramural vascular invasion (EMVI), threatened mesorectal fascia (MRF), or intersphincteric invasion. The TNT regimen consisted of induction chemotherapy (4 cycles of XELOX) followed by concurrent chemoradiotherapy (45–50.4 Gy pelvic radiation with capecitabine), with surgery performed 6–8 weeks after completion.

Conventional neoadjuvant chemoradiotherapy was administered to patients with locally advanced tumors (clinical T3/T4 or node-positive disease) who did not exhibit the high-risk features listed above. These patients received concurrent chemoradiotherapy (same radiation dose with capecitabine) followed by surgery after 6–8 weeks. The final decision regarding neoadjuvant regimen was made on a case-by-case basis by the MDT, taking into account not only tumor characteristics but also patient performance status and comorbidities.

Intestinal obstruction was defined as partial (incomplete) obstruction based on the following criteria: (1) colonoscopy revealed that the tumor completely obstructed the bowel lumen, preventing passage of the endoscope; (2) patients presented with clinical symptoms of obstructed defecation, including difficulty passing stool and reliance on laxatives for bowel movements; and (3) patients remained able to pass flatus and at least some stool, indicating incomplete obstruction. Patients with complete obstruction requiring emergency intervention or presenting with peritonitis, bowel perforation, or hemodynamic instability were excluded from this study.

The decision to create a diverting stoma was made at the discretion of the operating surgeon based on intraoperative findings. In our center, diverting stoma was not routinely performed. Indications for stoma formation included intraoperative concerns about anastomotic integrity, such as: (1) significant edema or dilation of the proximal bowel; (2) poor bowel preparation with substantial fecal residue; (3) compromised blood supply to the anastomotic site; (4) a very low anastomosis (≤5 cm from the anal verge); or (5) technical difficulties or tension during anastomosis construction. Patients without these findings typically proceeded without diversion.

### Definition and diagnosis of AL

In this study, AL referred exclusively to early leaks occurring within 30 days after surgery. AL was defined as a structural defect at the intestinal anastomosis creating communication between the intraluminal and extraluminal spaces, including adjacent pelvic abscesses. All cases were classified according to the International Study Group of Rectal Cancer (ISREC) criteria: Grade A (asymptomatic, requiring no intervention), Grade B (clinically apparent but manageable with non-operative treatments like antibiotics or percutaneous drainage), and Grade C (severe leaks necessitating reoperation) (2). Diagnostic confirmation was achieved through digital rectal examination (DRE), endoscopic evaluation, or radiographic imaging.

### Statistical analysis

All statistical analyses were performed using R version 4.5.1. Continuous non-normally distributed variables were expressed as median and interquartile range (IQR) and compared with the Wilcoxon rank-sum test. Categorical variables were expressed as frequencies (%), with ordinal categorical variables analyzed using the Kruskal-Wallis test and nominal categorical variables assessed using Pearson’s chi-square test or Fisher’s exact test. Variables with p < 0.1 in univariate analyses were included in multivariate logistic modeling. A backward stepwise logistic regression was employed to identify independent predictors of AL, with model performance evaluated using Nagelkerke’s pseudo R² and the concordance index (C-Statistic). Key predictors were further assessed using receiver operating characteristic (ROC) curve analysis. The final model was validated through calibration and clinical utility assessments. Calibration was evaluated using bootstrap-corrected calibration curves (1000 iterations), Brier score, and Hosmer-Lemeshow test (risk decile grouping). Clinical utility was analyzed via decision curve analysis (DCA) to quantify net benefit versus “treat-all/none” strategies. All statistical tests were two-sided, and p < 0.05 indicated statistical significance.

## Results

### Study participants

**Figure 1:**
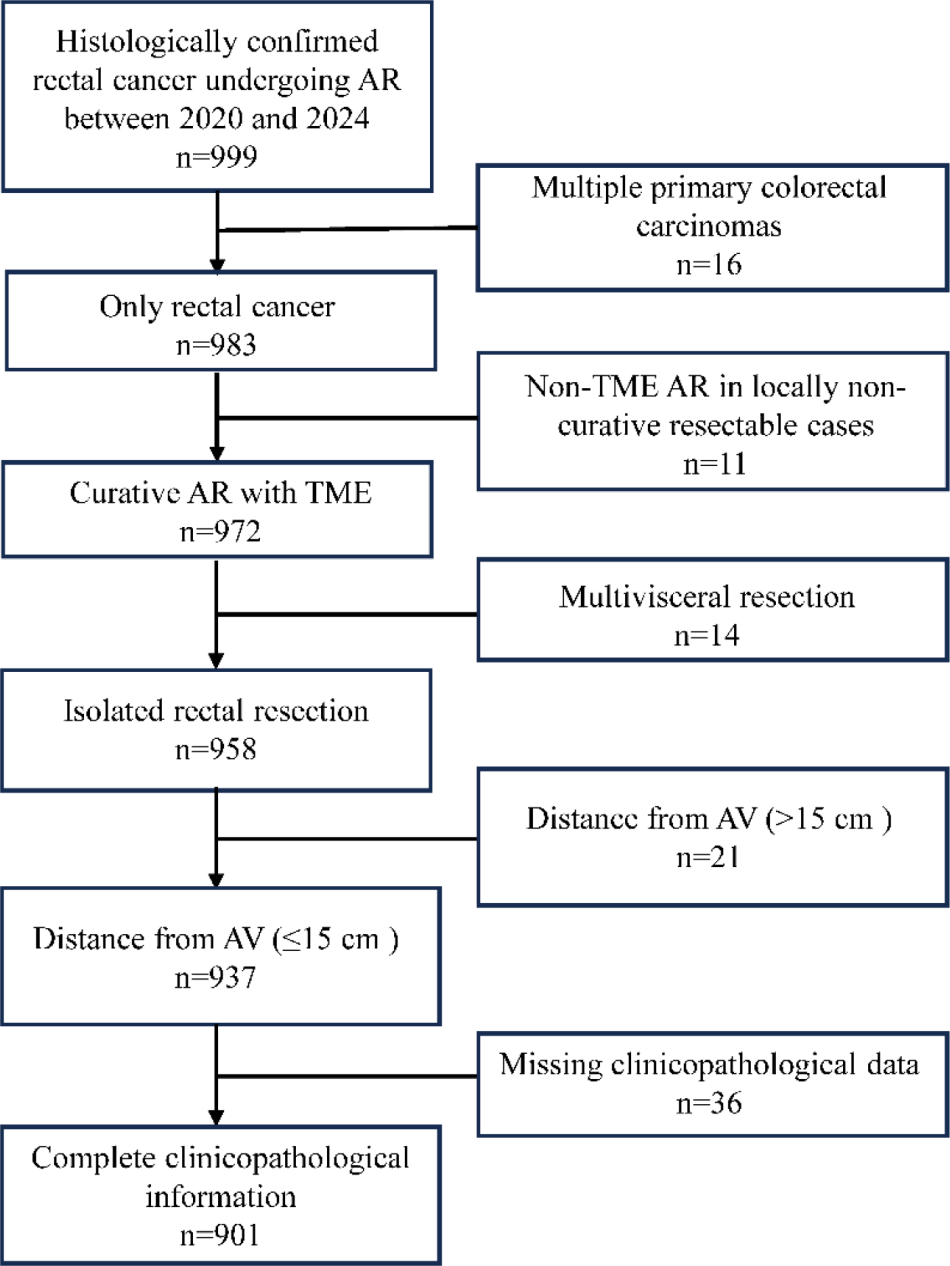
Flowchart of patient selection for rectal cancer AR cohort. AR, anterior resection; AV, anal verge. Missing clinicopathological data refers to the absence of multiple key preoperative and intraoperative clinical variables, not merely the lack of pathological factors.

Of the 999 patients who underwent rectal cancer resection during the study period, 901 met the inclusion criteria and were included in the final analysis. The detailed patient selection process is presented in **Figure 1**.

### Clinicopathological characteristics associated with AL after rectal cancer surgery

**Table 1** showed that this analysis of 901 patients revealed significant differences in preoperative characteristics between those without AL (n = 821) and those with AL (n = 80). The AL group had a higher proportion of males (10.5% vs 6.6%, p = 0.044), tumors located ≤ 5 cm from the AV (≤ 5 cm: 14.5% vs 9.0% vs 7.1%, p = 0.033), NACRT administration (22.3% vs 7.1%, p < 0.001), and intestinal obstruction (21.7% vs 8.2%, p = 0.002) before surgery. No significant differences were observed in age, ASA classification, BMI distribution, or preoperative albumin level (p > 0.1 for all).

**Table 1:** Comparative analysis of preoperative characteristics by AL status.

Characteristic	No AL (n = 821)	AL (n = 80)	P value
Age (year-old)	62.0 (55.0-69.0)	64.0 (55.8-68.0)	0.581
Gender			0.044
Female	352 (93.4%)	25 (6.6%)	
Male	469 (89.5%)	55 (10.5%)	
ASA			0.280
≤ II	757 (91.4%)	71 (8.6%)	
≥ III	64 (87.7%)	9 (12.3%)	
BMI (kg/m^2^)			0.487
< 18.5	53 (88.3%)	7 (11.7%)	
18.5-24	432 (92.1%)	37 (7.9%)	
> 24	336 (90.3%)	36 (9.7%)	
Distance from AV (cm)			0.033
≤ 5	112 (85.5%)	19 (14.5%)	
6-10	292 (91.0%)	29 (9.0%)	
> 10	417 (92.9%)	32 (7.1%)	
NACRT			< 0.001
No	741 (92.9%)	57 (7.1%)	
Yes	80 (77.7%)	23 (22.3%)	
Preoperative albumin (g/L)			0.157
< 30	7 (77.8%)	2 (22.2%)	
≥ 30	814 (91.3%)	78 (8.7%)	
Intestinal obstruction			0.002
No	785 (91.8%)	70 (8.2%)	
Yes	36 (78.3%)	10 (21.7%)	

AL, anastomotic leakage; ASA, American Society of Anesthesiologists; BMI, body mass index; AV, anal verge; NACRT, neoadjuvant chemoradiotherapy.

P-values in bold are statistically significant (P < 0.05).

**Table 2** revealed intraoperative/postoperative differences between non-AL and AL cohorts. Patients with AL had longer operative times (≥ 180 min: 13.8% vs 8.2%, p = 0.046), significantly fewer diverting stomas (9.6% vs 3.0%, p = 0.030), and higher rates of ISR (30.4% vs 8.3%, p < 0.001). Postoperative pain, as assessed by the NRS, was significantly greater in the AL group than in the non-AL group, with median scores of 4.0 (IQR 2.8–5.0) versus 2.0 (IQR 1.0–3.0), respectively (p < 0.001). No significant differences existed in blood loss, surgical approach, anastomotic technique, transanal drainage, or lymph node yield (all p > 0.1).

**Table 2:** Association of perioperative variables with AL.

Characteristic	No AL (n = 821)	AL (n = 80)	P value
Operative time (min)			0.046
< 180	721 (91.8%)	64 (8.2%)	
≥ 180	100 (86.2%)	16 (13.8%)	
Blood loss (mL)			0.787
< 100	444 (91.4%)	42 (8.6%)	
≥ 100	377 (90.8%)	38 (9.2%)	
Surgical approaches			0.215
Open AR	392 (89.9%)	44 (10.1%)	
Laparoscopic AR	429 (92.3%)	36 (7.7%)	
Anastomotic techniques			0.227
End-to-end	487 (90.2%)	53 (9.8%)	
Side-to-end	334 (92.5%)	27 (7.5%)	
Diverting stoma			0.030
No	725 (90.4%)	77 (9.6%)	
Yes	96 (97.0%)	3 (3.0%)	
Transanal drainage tube			0.124
Yes	392 (92.7%)	31 (7.3%)	
No	429 (89.7%)	49 (10.3%)	
Total lymph node yield			0.563
< 12	143 (89.9%)	16 (10.1%)	
≥ 12	678 (91.4%)	64 (8.6%)	
ISR			< 0.001
No	805 (91.7%)	73 (8.3%)	
Yes	16 (69.6%)	7 (30.4%)	
NRS pain scores	2.0 (1.0-3.0)	4.0 (2.8-5.0)	**< 0.001**

AL, anastomotic leakage; AR, anterior resection; ISR, intersphincteric resection. NRS, numerical rating scale.

P-values in bold are statistically significant (P < 0.05).

Pathological differences between the non-AL and AL groups are summarized in **Table 3**. Patients with AL had larger tumors (≥ 5 cm: 11.6% vs 7.3%, p < 0.001) and higher rates of distant metastasis (cM1: 15.6% vs 8.3%, p = 0.031). No significant differences were found in pT stage, pN status, tumor deposits, pTNM staging, or inferior mesenteric lymph node involvement (all p > 0.1).

**Table 3:** Tumor characteristics stratified by AL status.

Characteristic	No AL (n = 821)	AL (n = 80)	P value
Tumor size (cm)			< 0.001
< 5	532 (92.7%)	42 (7.3%)	
≥ 5	289 (88.4%)	38 (11.6%)	
pT			0.108
Tis	23 (95.8%)	1 (4.2%)	
T1	49 (98.0%)	1 (2.0%)	
T2	136 (94.4%)	8 (5.6%)	
T3	509 (90.1%)	56 (9.9%)	
T4	104 (88.1%)	14 (11.9%)	
pN			0.422
N0	506 (91.3%)	48 (8.7%)	
N1	212 (92.2%)	18 (7.8%)	
N2	103 (88.0%)	14 (12.0%)	
cM			0.031
M0	756 (91.7%)	68 (8.3%)	
M1	65 (84.4%)	12 (15.6%)	
Tumor deposit			0.981
No	627 (91.1%)	61 (8.9%)	
Yes	194 (91.1%)	19 (8.9%)	
pTNM			0.154
0	23 (92.0%)	2 (8.0%)	
I	146 (94.2%)	9 (5.8%)	
II	268 (90.2%)	29 (9.8%)	
III	319 (91.9%)	28 (8.1%)	
IV	65 (84.4%)	12 (15.6%)	
Inferior mesenteric lymph nodes			0.308
Negative	781 (91.3%)	74 (8.7%)	
Positive	40 (87.0%)	6 (13.0%)	

AL, anastomotic leakage; pTNM classification: p, pathological (postoperative histological confirmation); c, clinical (imaging confirmation); Tis, carcinoma *in situ*; T, tumor invasion depth; N, regional lymph node involvement; M, distant metastasis.

P-values in bold are statistically significant (P < 0.05).

### Predictive model for AL

**Figure 2:**
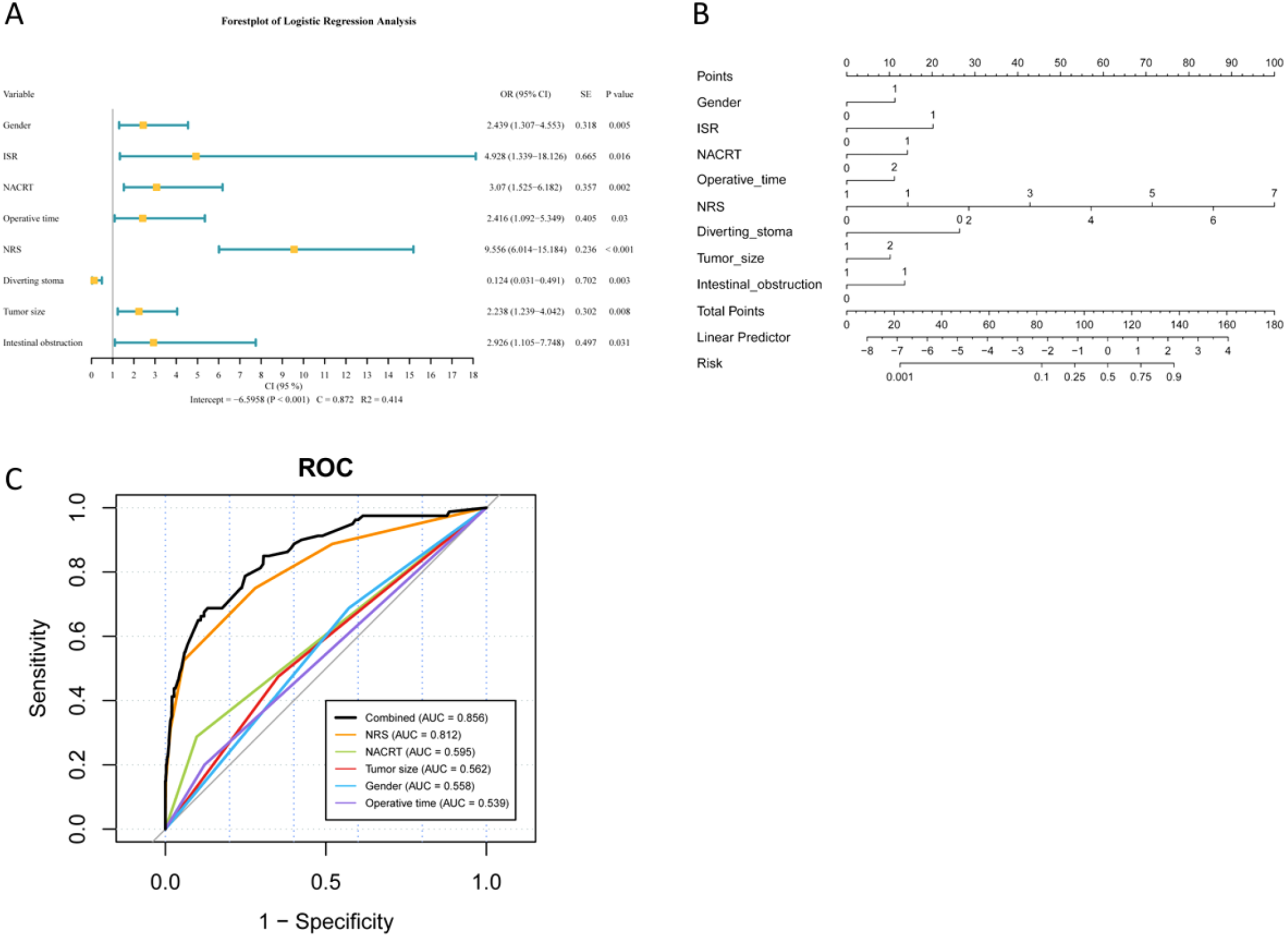
Predictive model for AL in rectal cancer surgery. **(A)** Significant predictors of AL. Forest plot of multivariable logistic regression results. **(B)** AL risk nomogram. Clinical tool integrating eight independent predictors. **(C)** ROC analysis of top predictors. Performance comparison of individual and combined variables. AL, anastomotic leakage; ISR, intersphincteric resection; NACRT, neoadjuvant chemoradiotherapy; NRS, Numerical Rating Scale; ROC, receiver operating characteristic.

Candidate predictors of AL—including preoperative characteristics, intraoperative variables, and early postoperative factors (with the NRS pain score recorded prior to leakage diagnosis), but excluding pathological variables—were first examined by univariable analysis. Variables with a significance level of p < 0.10 were subsequently entered into the multivariable logistic regression, which identified eight independent predictors of AL (**Figure 2A**). Postoperative NRS pain scores demonstrated the strongest association (OR = 9.556; 95% CI, 6.014-15.184; p < 0.001), followed by ISR (OR = 4.928; 95% CI, 1.340-18.126; p = 0.016) and NACRT (OR = 3.070; 95% CI, 1.525-6.182; p = 0.002), intestinal obstruction (OR = 2.926; 95% CI, 1.105-7.748; p = 0.031), operative time (OR = 2.416; 95% CI, 1.092-5.349; p = 0.030), and tumor size (OR = 2.238; 95% CI, 1.239-4.042; p = 0.008). Protective effects were observed for diverting stoma (OR = 0.124; 95% CI, 0.031-0.491; p = 0.003) and female gender (OR = 0.410; 95% CI, 0.220-0.765; p = 0.005). The model exhibited excellent discrimination (C-statistic = 0.872) and explained 41.4% of variance (R² = 0.414).

A predictive nomogram was developed, derived from multivariate logistic regression analysis, to estimate the risk of rectal AL (**Figure 2B**). The nomogram incorporates the eight independent factors identified above, assigning each variable a score proportional to its contribution to overall risk, with the total points corresponding to the predicted probability of AL. This tool enables individualized risk stratification and offers a visual, quantitative aid to guide perioperative clinical decision-making.

The predictive performance of the top five risk variables for AL after rectal cancer surgery among eight independent variables—including NRS, NACRT, tumor size, gender, and operative time—was assessed using ROC analysis. Notably, NRS exhibited the highest individual predictive value (AUC = 0.812), with AUCs ranging from 0.539 to 0.595 for the remaining variables. A combined model incorporating all five variables demonstrated the best overall performance (AUC = 0.856). **Figure 2C** illustrates the ROC curves of these variables, allowing visual comparison of their diagnostic accuracy.

### Validation of the combined AL prediction model: calibration and clinical applicability

**Figure 3:**
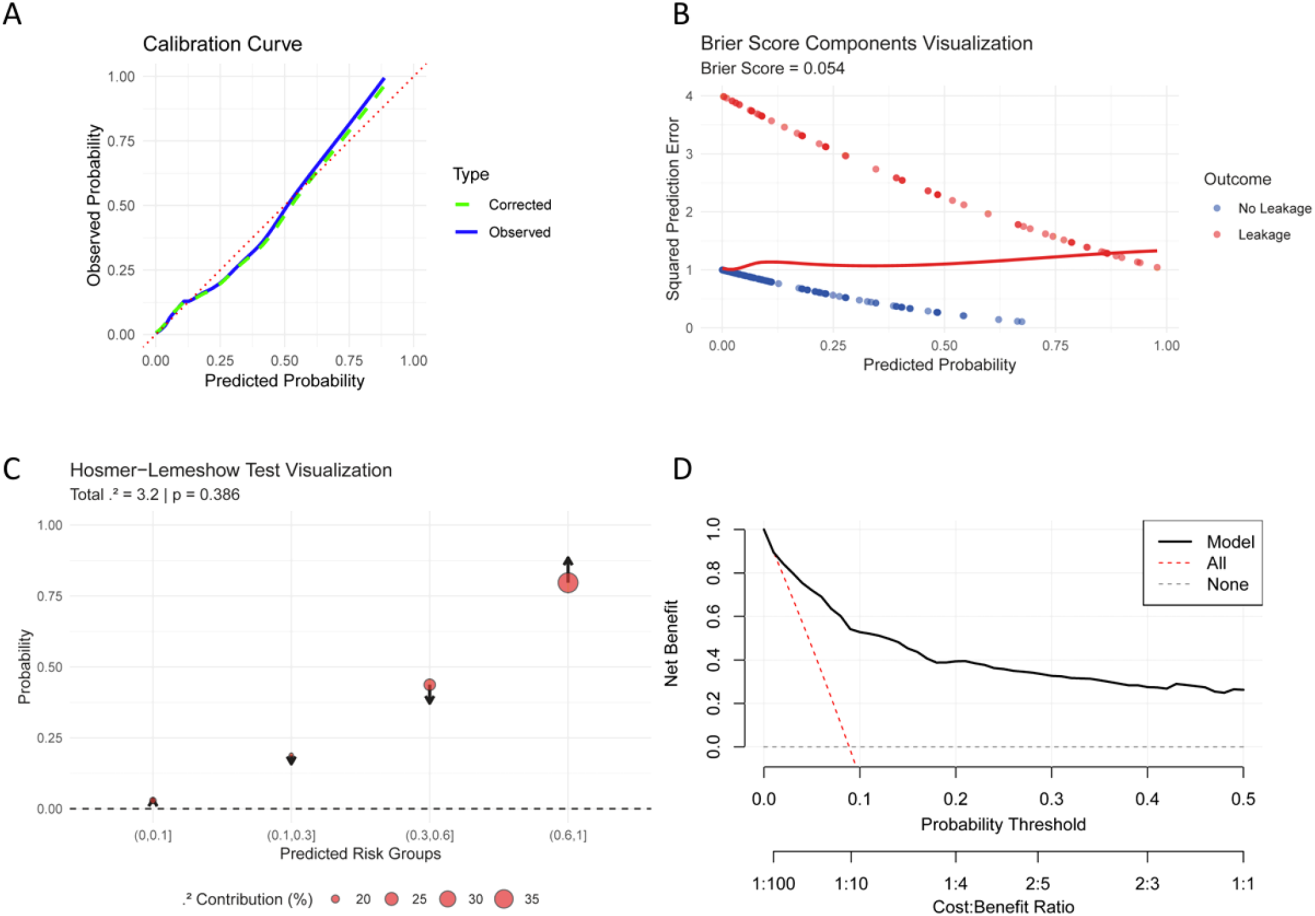
Validation of the AL prediction model. **(A)** Calibration curve. Agreement between predicted and observed AL probabilities. **(B)** Brier score decomposition. Visualization of prediction error components. Grading Scale: < 0.05: Excellent calibration (minimal prediction error); 0.05-0.10: Acceptable for clinical use (moderate error); > 0.10: Requires improvement (substantial miscalibration). Range: 0 = perfect prediction; 0.25 = worst possible. **(C)** Hosmer-Lemeshow test. Observed vs. expected event frequencies across risk deciles. The model showed good calibration (p > 0.05). **(D)** Decision curve analysis. Net benefit of the model compared to treat-all and treat-none strategies across probability thresholds. AL, anastomotic leakage.

Based on the calibration curve in **Figure 3A**, the predicted probabilities from the combined model align closely with the observed outcomes, indicating good calibration. The corrected line closely follows the ideal diagonal, suggesting that the model’s predicted risk of AL accurately reflects actual probabilities. This supports the reliability of the combined model in clinical prediction. In addition to the calibration curve, the predictive performance of the combined model was further evaluated using the Brier score, Hosmer-Lemeshow test, and decision curve analysis. In our cohort of 901 rectal cancer patients, the model demonstrated good calibration with a Brier score of 0.054, indicating acceptable accuracy for clinical use (**Figure 3B**). The modified calibration plot visualizes the Hosmer-Lemeshow test, with arrows showing discrepancies between predicted and observed outcomes and bubble sizes reflecting each group’s contribution to the χ² statistic (p = 0.39; **Figure 3C**). The largest deviation was noted in the 30-60% risk group, but its impact was limited due to a small sample size (n = 43). DCA showed that the model offered greater net benefit than “treat all” or “treat none” strategies across threshold probabilities of 8% to 35%. At a 20% threshold, the model could reduce unnecessary interventions in 15 out of 100 patients (**Figure 3D**).

### Predictors of Grade C AL

Based on ISREC criteria applied to 901 patients, AL occurred in 80 cases (8.9%), with severity distributed as follows: Grade A in 12 patients (1.3%), Grade B in 26 (2.9%), and Grade C-the most severe subtype-in 42 patients (4.7%), constituting 52.5% (42/80) of all AL cases. The majority (821 patients, 91.1%) had no AL (**Table 4**).

**Table 4:** Grading system for AL.

Characteristic	Number	Percentage
Total	901	100%
No AL	821	91.1%
Total AL	80	8.9%
Grade A	12	1.3%
Grade B	26	2.9%
Grade C	42	4.7%

AL, anastomotic leakage; Anastomotic leak severity was graded according to the International Study Group of Rectal Cancer criteria.

Among the 80 patients who developed anastomotic leakage (AL), we aimed to identify factors associated with Grade C AL. To identify factors associated with Grade C among AL patients, we further evaluated eight clinically significant characteristics previously identified by multivariable logistic regression. Significant differences were observed between minor (Grade A–B, n = 38) and major (Grade C, n = 42) AL groups (**Table 5**). Patients with Grade C AL reported higher postoperative NRS pain scores (median 4.5 [IQR 3.0-5.0] vs 3.0 [2.0-4.0], p < 0.001) and received NACRT more frequently (73.9% vs 43.9%, p = 0.015) compared to those with Grade A-B AL. No significant differences were found in gender distribution, operative time, diverting stoma, ISR frequency, tumor size, or preoperative intestinal obstruction (all p > 0.05).

**Table 5:** Comparison of clinicopathological characteristics by AL severity.

Characteristic	Grade A-B (n = 38)	Grade C (n = 42)	P value
NRS criteria	3.0 (2.0-4.0)	4.5 (3.0-5.0)	**< 0.001**
NACRT			0.015
No	32 (56.1%)	25 (43.9%)	
Yes	6 (26.1%)	17 (73.9%)	
Gender			0.952
Female	12 (48.0%)	13 (52.0%)	
Male	26 (47.3%)	29 (52.7%)	
ISR			0.294
No	36 (49.3%)	37 (50.7%)	
Yes	2 (28.6%)	5 (71.4%)	
Operative time (min)			0.179
< 180	28 (43.8%)	36 (56.3%)	
≥ 180	10 (62.5%)	6 (37.5%)	
Diverting stoma			
No	36 (46.8%)	41 (53.2%)	
Yes	2 (66.7%)	1 (33.3%)	
Tumor size (cm)			0.593
< 5	21 (50%)	21 (50.00%)	
≥ 5	17 (44.7%)	21 (55.3%)	
Intestinal obstruction			0.866
No	33 (47.1%)	37 (52.9%)	
Yes	5 (50%)	5 (50%)	

AL, anastomotic leakage; ISR, intersphincteric resection; NACRT, neoadjuvant chemoradiotherapy; NRS, numerical rating scale.

P-values in bold are statistically significant (P < 0.05).

### Predictive model and calibration for Grade C AL

**Figure 4:**
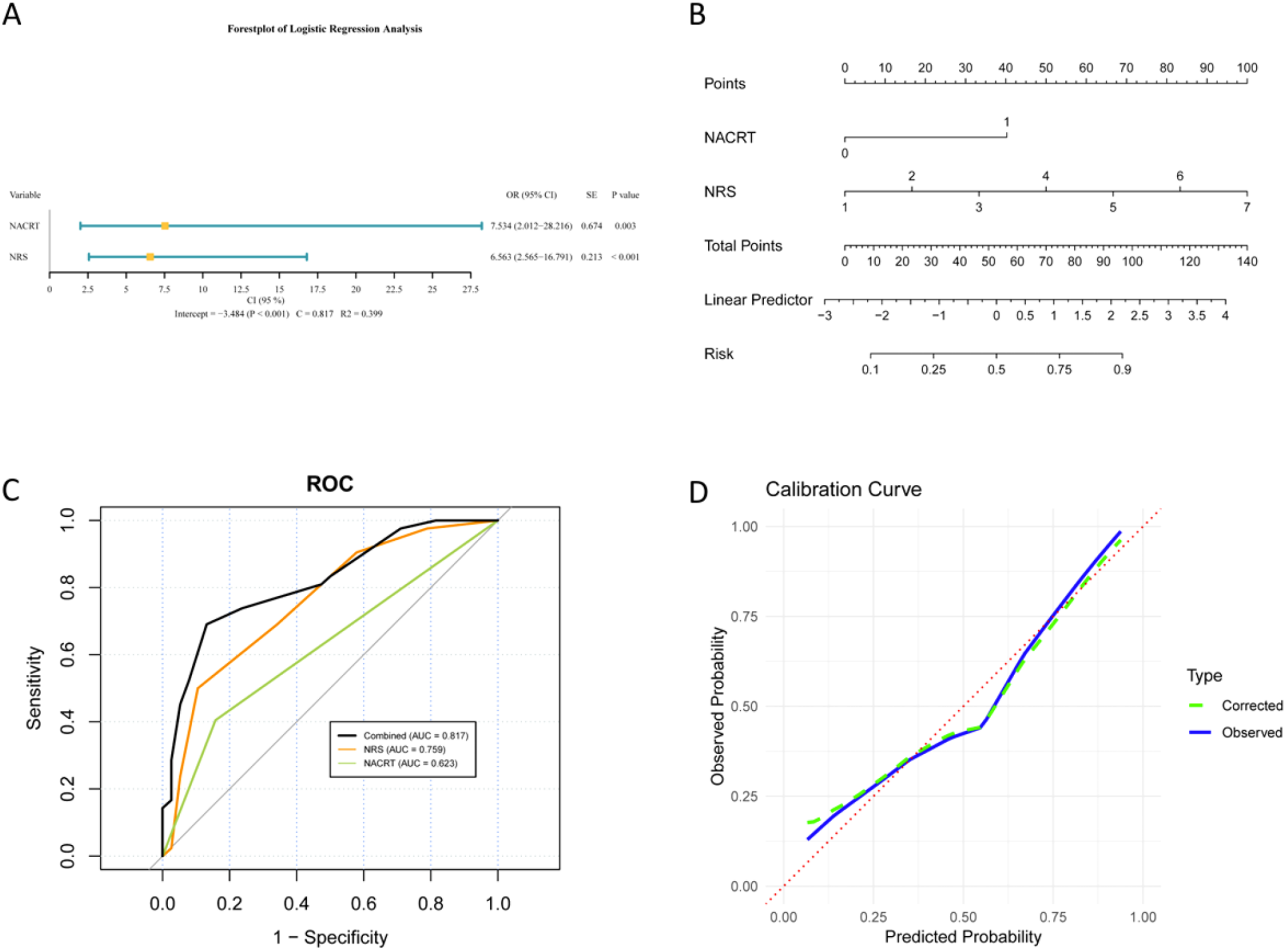
Predictive model for severe (Grade C) AL. **(A)** Multivariable predictors. Forest plot showing NACRT and NRS as independent risk factors. **(B)** Risk nomogram. Clinical tool converting NACRT and NRS scores to probability points. **(C)** ROC analysis. Discriminatory performance of the combined model versus individual predictors. **(D)** Calibration curve. Agreement between predicted and observed Grade C AL frequencies. AL, anastomotic leakage; NACRT, neoadjuvant chemoradiotherapy; NRS, Numerical Rating Scale; ROC, receiver operating characteristic.

**Figure 4A** presented that the multivariable logistic regression analysis identified NACRT and NRS as significant independent predictors for Grade C leakage. NACRT demonstrated an OR of 7.534 (95% CI, 2.012-28.216; p = 0.003), while NRS had an OR of 6.563 (95% CI, 2.565-16.791; p < 0.001). The model exhibited strong discriminatory power (C-statistic = 0.817) and explained variance (R² = 0.399).

A predictive nomogram integrating NACRT and NRS was developed to quantify individual risk probabilities for Grade C leakage, translating these predictors into a points system corresponding to probabilities (**Figure 4B**). The model’s performance was further validated through ROC analysis, showing excellent discrimination for Grade C leakage (AUC = 0.817), which surpassed the individual predictive capacity of NACRT (AUC = 0.623) or NRS alone (AUC = 0.759; **Figure 4C**). Calibration curve analysis confirmed robust agreement between predicted probabilities and observed frequencies across the risk spectrum (**Figure 4D**). These results collectively indicate that NACRT and NRS are robust risk factors for severe anastomotic leakage, with the combined model providing high predictive accuracy and reliable calibration for clinical risk stratification.

## Discussion

This single-center retrospective study systematically evaluated clinical and pathological predictors of AL following rectal cancer surgery, with a particular focus on identifying risk factors for Grade C AL, the most severe and clinically consequential subtype. Our findings demonstrated that postoperative NRS pain scores and NACRT were key independent predictors of AL, particularly Grade C leaks, while diverting stoma placement exerts a protective effect. These results align with and extend previous research, offering clinically actionable insights for risk stratification and perioperative management (9, 12).

### NRS as an early warning sign for AL

The strong association between elevated postoperative NRS pain scores and AL risk suggests that pain severity may serve as an early warning sign of anastomotic compromise, consistent with Boström et al. (12). In our analysis, we used the NRS score recorded on the day prior to AL diagnosis to test whether pain precedes clinical deterioration. While this specific time point cannot be known prospectively, our findings confirm that pain is a prodromal symptom of AL. Clinically, this means that any unexplained escalation in pain—whether on postoperative day 2, 3, or 4—should raise suspicion and prompt further evaluation. Patients with moderate-to-severe pain (NRS ≥ 4) without an alternative explanation warrant heightened surveillance, as they may be at increased risk for AL, particularly Grade C leaks.

In this study, NRS was evaluated as an early warning sign for impending AL. Several aspects warrant discussion. First, regarding validity and bias: For AL patients, we used the score from the day before diagnosis; for non-AL patients, the highest postoperative score served as comparator. This retrospective asymmetry may introduce lead-time bias, and pain subjectivity plus variable analgesic use could confound results. Second, regarding clinical applicability: Since the exact “day before diagnosis” cannot be known prospectively, the clinical value lies not in a single score but in the principle that any unexplained escalation in pain should raise suspicion and prompt further investigation. Third, NRS is a risk stratification tool, not a diagnostic test—it identifies who needs closer monitoring, complementing rather than replacing CT or CRP.

Beyond clinical symptoms, emerging evidence suggests that preoperative systemic inflammation may also play a role in AL pathogenesis. Golshani et al. found that a high modified Glasgow Prognostic Score (mGPS=2: OR 4.11) and elevated C-reactive protein-to-albumin ratio (CAR>0.36: OR 2.25) were associated with increased AL risk after rectal cancer surgery, although predictive accuracy was modest (AUC up to 0.73) (14). These findings support the potential value of integrating inflammatory markers with early postoperative clinical signs such as pain in future risk stratification models. Unfortunately, due to the non-routine measurement of CRP in our center during the study period, we were unable to validate these inflammatory indices in our cohort. Future prospective studies should aim to integrate both dynamic early warning signs (e.g., pain trajectories) and preoperative inflammatory biomarkers (e.g., CAR, mGPS) to develop more comprehensive and accurate risk stratification models for AL.

### Other key findings and clinical implications

NACRT has been identified as an independent predictor of AL, particularly for severe (Grade C) leaks. While the oncological benefits of NACRT are well established, its impact on surgical healing remains clinically significant. The study by Zaborowski et al. (10) reported a paradoxical increase in AL rates even among patients achieving pathological complete response (pCR), suggesting that treatment-induced tissue changes—such as fibrosis and microvascular compromise—contribute to impaired anastomotic healing and creating a high-risk environment for AL (15, 16).

These findings highlighted the need for tailored risk assessment in NACRT-treated patients. Postoperative monitoring should be intensified, particularly in pCR cases, where AL risk may be underestimated. Early warning signs, such as elevated pain scores or inflammatory markers, should prompt timely intervention. Surgical strategies, including selective stoma use or reinforced anastomotic techniques, may mitigate leakage risk in this vulnerable population. A balanced approach—optimizing both oncological outcomes and surgical safety—is essential in rectal cancer management.

In our multivariable analysis, male, ISR, and longer operative time (≥ 180 min) were identified as significant risk factors for predicting AL. Quan et al.’s reported that postoperative anastomotic leakage in low rectal cancer is affected by multiple variables, including patient sex, tumor-to-anal verge distance, and surgical duration (17). The possible reasons for the higher risk of anastomotic leakage in male patients after rectal surgery include anatomical differences, such as a narrower and deeper pelvis, which makes surgical access more difficult and increases the risk of intraoperative trauma. These factors may impair local blood supply and healing at the anastomotic site, leading to a higher incidence of leakage. Beyond anatomical factors, emerging evidence suggests that sex hormones may influence anastomotic leakage risk. A nationwide study by Rutegård et al. (18) found that women taking estrogen-increasing drugs before surgery had a significantly lower risk of leakage, suggesting a protective effect of estrogen on healing. A complementary study by the same group (19) found that men receiving androgen deprivation therapy showed a trend toward lower leakage risk (OR: 0.70), though this was not statistically significant. These findings suggest that the higher leakage risk in men may result from both lack of estrogen protection and potential harmful effects of androgens, offering a biological explanation beyond pelvic anatomy.

ISR is a sphincter-preserving surgical technique used in ultralow rectal cancer, especially when the tumor is very close to the anus but sphincter function can still be preserved. The technical complexity of ISR, combined with increased intraluminal pressure, contributes to the inherent fragility of ultralow anastomoses. Relevant studies have shown that AL following ISR for ultralow rectal cancer may lead to adverse outcomes such as chronic anastomotic stricture and permanent stoma, particularly in cases complicated by anastomotic separation (20, 21). Degiuli et al. conducted a multicenter study analyzing rectal cancer surgeries across 24 Italian hospitals (7). Their research identified prolonged surgical duration as a significant contributor to anastomotic leakage risk. This association highlights two critical clinical implications: first, the importance of surgical team training to optimize efficiency without compromising safety; second, the necessity for enhanced postoperative surveillance in cases where extended operative times are unavoidable. The study underscores that while some time-extending factors like complex anatomy may be unavoidable, systematic efforts to streamline procedures could meaningfully reduce leakage rates. These findings advocate for a balanced approach where technical precision is maintained while minimizing unnecessary delays that may compromise anastomotic integrity.

Our study also identified larger tumor size (≥ 5 cm) and intestinal obstruction as significant risk factors for AL following rectal cancer surgery, which aligns with the findings of Matsuzaki et al., who reported that tumor obstruction and tumor size were key predictors of AL in laparoscopic anterior resection for rectal cancer (22). Large tumors may increase AL risk through three key mechanisms: (1) More surgical trauma from extensive dissection, reducing blood flow to the healing area; (2) Wider surgical connections that heal poorly compared to smaller joins; (3) Large tumors causing bowel obstruction lead to proximal bowel dilation, edema, and compromised blood flow, impairing anastomotic healing and increasing leakage risk.

### Protective strategy and risk mitigation

A randomized trial demonstrated that rectal cancer patients with low anastomoses (< 8 cm) receiving diverting ileostomies had significantly lower leakage rates (5.8% vs 16.3%) and reoperation rates compared to non-diverted patients. The protective effect was most pronounced in male patients and those with ultralow (< 6 cm) anastomoses, supporting routine stoma use in these high-risk cases (23). Multiple meta-analyses found diverting stomas after rectal cancer surgery significantly reduce anastomotic leaks and reoperations without increasing mortality (24, 25). The protective role of diverting stomas reinforces existing evidence that fecal diversion reduces AL-related morbidity by mitigating septic complications. Our data suggest that stoma creation should be strongly considered in high-risk patients, particularly those with male, ISR, larger tumor size (≥ 5 cm), prolonged operative times (≥180 min), or NACRT exposure. Conversely, the absence of a stoma in such cases may significantly elevate AL risk, underscoring the need for individualized decision-making in surgical planning.

### Practical application of the nomogram

To demonstrate the clinical utility of the nomogram, consider a typical high-risk patient: a 65-year-old female who underwent ISR after NACRT, with a postoperative NRS pain score of 5 and an operative time of 100 min, without diverting stoma creation. Using the nomogram (**Figures 2B** and **4B**) for this patient profile, the nomogram generates a predicted AL score of approximately 105 points (corresponding to 40% leakage risk) and a Grade C-specific score of 118 points (indicating > 90% risk of severe leakage). This striking difference in risk levels-especially the extremely high chance of severe Grade C leakage- should prompt postoperative surveillance and early diagnostic interventions.

It is important to note that while the nomogram incorporates postoperative pain scores, the decision to create a diverting stoma must be made intraoperatively, before pain data are available. Therefore, the primary value of this nomogram lies in guiding early postoperative management. For patients identified as high-risk based on this model, we recommend enhanced monitoring protocols, including protocolized CT imaging between postoperative days 3–5 regardless of clinical symptoms, complemented by 8-hourly CRP measurements and continuous temperature monitoring.

This systematic application exemplifies how the nomogram transforms preoperative and intraoperative variables, combined with early postoperative pain assessment, into a quantitative risk tool that enables personalized postoperative surveillance. By aligning the intensity of monitoring with predicted failure risks, clinicians can optimize resource allocation while avoiding unnecessary interventions in lower-risk scenarios.

Several prediction models for AL after rectal cancer surgery have been developed in recent years. Notably, Rutegård et al. recently developed and validated a risk score specifically for the SELSA trial (Selective defunctioning Stoma Approach in low anterior resection for rectal cancer) (26, 27). Using a large Swedish nationwide registry cohort of 2,727 patients, they identified three key preoperative predictors: male sex, BMI >30 kg/m², and neoadjuvant radiotherapy. Each predictor contributed one point in a simple scoring system, where a score of 0–1 indicated a predicted leak risk ≤10%. Their model showed moderate discrimination (AUC 0.64) but a high negative predictive value (94.6%), making it suitable for ruling out low-risk patients who might safely avoid a defunctioning stoma in the upcoming SELSA trial.

Our study differs from the SELSA model in several important ways. First, the SELSA model was designed for intraoperative decision-making regarding stoma omission in low-risk patients, whereas our model aims to guide early postoperative management in high-risk patients. Second, the SELSA model relies exclusively on preoperative variables (sex, BMI, radiotherapy), while our model incorporates early postoperative factors, particularly the NRS pain score, which emerged as the strongest predictor. This allows our model to capture dynamic changes in a patient’s condition that static preoperative models cannot. Third, our model includes a broader range of predictors (e.g., ISR, operative time, tumor size, diverting stoma, intestinal obstruction) and provides risk estimates for both overall AL and Grade C severe leakage, enabling more nuanced risk stratification.

Despite these differences, both studies highlight the importance of male sex and neoadjuvant therapy as key risk factors. The SELSA model’s simple scoring system offers excellent clinical usability for preoperative risk screening, while our model contributes by demonstrating that postoperative pain adds significant predictive value for early warning. We believe these two approaches are complementary: the SELSA model helps decide who can safely avoid a stoma, while our model helps identify who needs enhanced monitoring after surgery. Future studies should explore integrating both static preoperative factors and dynamic postoperative variables into a comprehensive perioperative risk stratification pathway.

### Limitations and future directions

Several limitations warrant consideration. First, the retrospective design precludes causal inferences, and potential confounding factors (e.g., surgeon experience, anastomotic technique variations) may influence outcomes. Second, while NRS scores were systematically recorded, interobserver variability in pain assessment could introduce bias. Future prospective studies should standardize pain evaluation protocols. Third, while backward elimination was used to develop this clinically interpretable model, we acknowledge that this method has limitations. Although we used a liberal threshold (p < 0.10) and ensured clinical plausibility of the selected variables, more modern techniques such as LASSO regression could provide additional shrinkage and potentially improve external validity. Future studies with larger cohorts are encouraged to apply such methods to validate our findings. Fourth, while bootstrap resampling demonstrated good internal validity (optimism-corrected C-statistic: 0.852), the model was developed and validated using data from a single center without an external cohort. Multicenter validation of our predictive model is needed to confirm its robustness across diverse practice settings. Fifth, this study did not compare the predictive performance of our model with established diagnostic tools such as CT imaging or rectal examination. It should be noted that CT and rectal examination are primarily used for confirming AL, whereas our model is designed as a risk stratification tool to identify high-risk patients who may benefit from such diagnostic investigations. Future studies should explore how to best integrate our model with these diagnostic modalities to create a comprehensive clinical pathway—using the model for early risk assessment to trigger timely confirmatory testing. Sixth, as postoperative imaging was performed based on clinical suspicion rather than routine protocol, asymptomatic Grade A leaks may have been underdiagnosed. This could lead to misclassification of some patients as non-leak cases. However, our primary focus was on clinically significant leaks (Grades B and C) that require intervention, as these have the greatest impact on patient outcomes.

## Conclusion

In summary, this study identifies postoperative NRS pain scores and NACRT as key predictors of AL, particularly Grade C leaks, while reinforcing the protective role of diverting stomas. These findings advocate for enhanced postoperative monitoring in high-risk patients and support the integration of pain assessment into AL risk stratification models. Future research should explore whether targeted interventions—such as intensified surveillance, early reintervention—can reduce AL incidence and severity in vulnerable populations. By refining risk prediction and perioperative strategies, we may improve outcomes for rectal cancer patients undergoing curative resection.

## Data Availability

The raw data supporting the conclusions of this article will be made available by the authors, without undue reservation.
